# Brief potentially ictal rhythmic discharges on intraoperative electrocorticography predict a good outcome of focal cortical dysplasia after surgical resection: a case report

**DOI:** 10.1186/s42494-023-00131-6

**Published:** 2023-07-18

**Authors:** Sofía S. Sánchez-Boluarte, Wilfor Aguirre-Quispe, Manuel Herrera Aramburú, William O. Tatum, Walter De La Cruz Ramírez

**Affiliations:** 1 School of Medicine, Cesar Vallejo University, Av. Victor Larco, Trujillo, Trujillo 1770 Peru; 2 Departamento de Epilepsia, Instituto Nacional de Ciencias Neurológicas, Jr. Ancash 1271, Lima, Lima 15001 Peru; 3 Neurosciences, Clinical Effectiveness and Public Health Research Group, Universidad Científica del Sur, Panamericana Sur 19, Villa EL Salvador, Lima, Lima 15067 Peru; 4 Department of Neurology, Mayo Clinic, Mangurian Bldg., Fourth Floor, 4500 San Pablo Road, FL 32224 Jacksonville, USA

**Keywords:** Electrocorticography, Cortical dysplasia-focal epilepsy syndrome, Drug-resistant epilepsy, Frontal lobe epilepsy

## Abstract

**Background:**

Focal cortical dysplasia (FCD) is a common cause of drug-resistant epilepsy. Electroencephalography (EEG) biomarkers that predict good postoperative outcomes are essential for identifying patients with focal epilepsies.

**Case presentation:**

We report the case of a 21-year-old female with seizure onset at the age of 9, characterized by left-hand dystonic posturing and impaired awareness, which evolved to bilateral tonic-clonic seizures, evaluated in a neurological referral center in Lima, Peru. During 6-h video-EEG, interictal EEG revealing focal brief potentially ictal rhythmic discharges (BIRDs) over the right frontal central region, lasting less than 10 s. The ictal features were characterized by low-voltage fast activity over the same area. Brain magnetic resonance imaging (MRI) demonstrated a focal lesion of focal cortical dysplasia type II in the right frontal lobe. The patient underwent a lesionectomy guided by electrocorticography, which showed continuous polyspikes. BIRDs showing a brief burst of spikes lasting longer than 0.5 s, were also identified on intraoperative electrocorticography (ECoG) and helped define the extent of resection. The patient obtained an Engel Outcome Class IA at 6 years of follow-up.

**Conclusions:**

The atypical BIRDs on ECoG can be used as a prognostic biomarker for prolonged seizure-freedom outcome in patients with epilepsy. Additional reports are needed in developing countries with and without brain MRI lesions to advance outpatient presurgical evaluations despite limited resources.

## Background

Epilepsy surgery is being carried out more frequently in low- and middle-income countries; however, it is mainly limited to patients with temporal lobe epilepsy [1]. Reports of epilepsy surgery in Peru have been limited to case series [2]. Frontal lobe epilepsy is the second most common type of focal epilepsy with poor outcomes due to high connectivity and network complexity, with focal cortical dysplasia (FCD) being one of the leading causes of drug-resistant epilepsy [2–4]. The lack of resources in Peru results in diverse challenges in presurgical evaluation, limited access to electroencephalography (EEG) machines, limited number of qualified technologists, and inadequate infrastructure, making it challenging to perform prolonged video-EEG monitoring. In selected cases, intraoperative electrocorticography (ECoG) is an indispensable tool for surgical decision-making [2].

Electrophysiological biomarkers, such as focal brief potentially ictal rhythmic discharges (BIRDs) [1] characterized by a sharply contoured delta activity and low-voltage ictal fast activity (LVFA), are crucial for identifying the seizure-onset zone in patients with neuronal migration disorders [2, 3, 5]. In this case report, we describe a patient with FCD who showed atypical BIRDs [1] on ECoG and scalp EEG. This patient became seizure-free following a surgical resection.

## Case presentation

A 21-year-old right-handed female with drug-resistant focal epilepsy was admitted to the epilepsy unit of the National Institute of Neurological Sciences in Lima-Perú with convulsive status epilepticus. The patient’s seizure onset occurred at the age of 9 without any epilepsy risk factors and the seizures were characterized by daily sudden-onset left-hand dystonic posturing with impaired awareness evolving to bilateral tonic-clonic seizures. Neurological examination revealed mild weakness in the left upper extremity and slight left hyperreflexia. The seizures persisted despite treatment with valproate, carbamazepine, and clobazam. She finally underwent presurgical assessment.

**Fig. 1:**
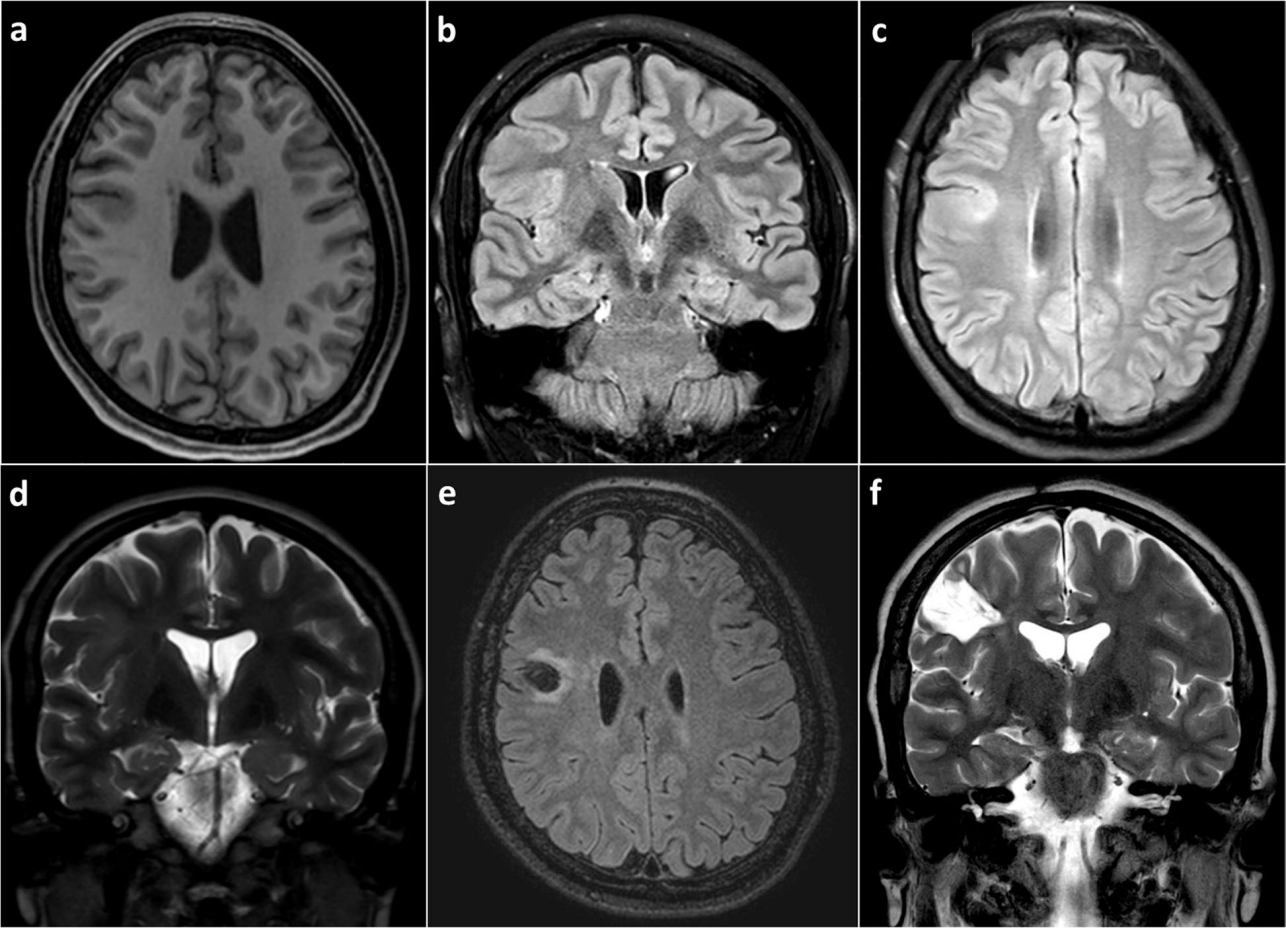
Brain MRI showed cortical thickening and loss of corticosubcortical differentiation in the premotor area on axial T1 (**a**), coronal T2-FLAIR (**b**), axial T2-FLAIR (**c**), and coronal T2 (**d**) images. Brain MRI performed after the procedure showed complete resection of the lesion on axial T2-FLAIR (**e**) and coronal T2-weighted (**f**) images

**Fig. 2:**
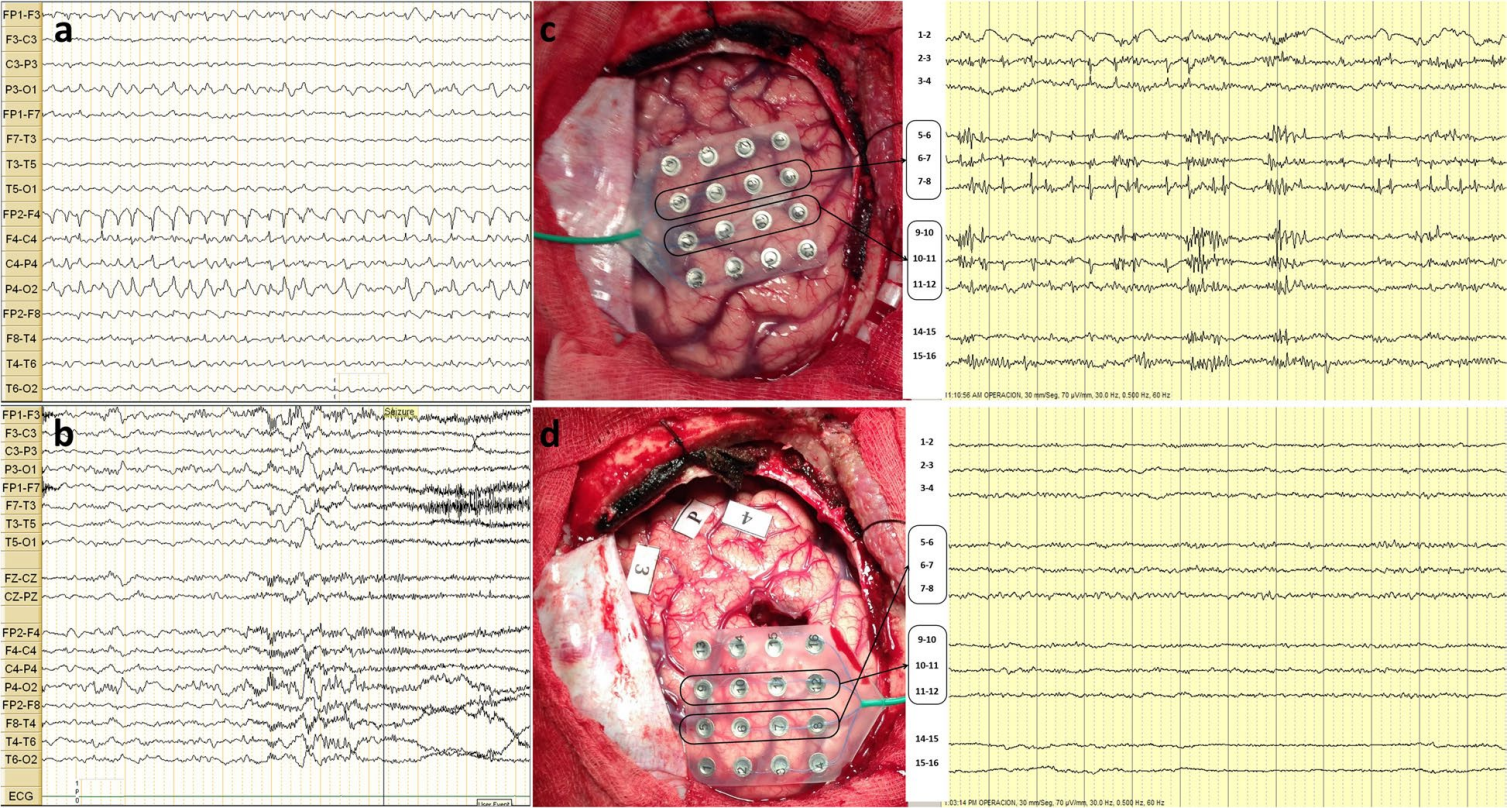
**a** Scalp EEG showed brief 2.5-Hz potentially ictal periodic discharges, with a maximal at F4 within a bilateral parasagittal field. **b** Ictal EEG showed diffuse low-voltage fast activity. **c** Intra-operative electrocorticography showed frequent polyspikes present diffusely and focal atypical BIRDs over a 16-channel 4 × 4 subdural grid placed over the right premotor cortex. **d** Electrocorticography after lesion resection showed no evidence of ongoing epileptiform activity. The labels on the photograph do not represent a particular area

A 3-T brain magnetic resonance imaging (MRI) with an epilepsy protocol revealed a right frontal lesion of FCD involving the premotor area (Fig. 1). The 6-h video-EEG showed focal BIRDs characterized by a sharply contoured rhythmic delta activity, with a maximum at F4 within a bilateral parasagittal field (with right-sided predominance), lasting less than 10 s at a frequency of 2.5-3 Hz. Ictal EEG was characterized by an abrupt onset of low-voltage fast activity maximal over the F4-C4 electrode derivation (Fig. 2). Blood tests, including complete blood count, electrolytes, thyroid profile, and liver and kidney function tests, were normal.

Neuropsychological evaluation revealed right frontotemporal lobe dysfunction due to compromise of executive functions, specifically inhibitory control, visual span, phonological fluency, semantics, and visual memory (visual memory reserve 26.6%), language dominance was localized in the left hemisphere. These results were consistent with the EEG and imaging findings. Intraoperative electrocorticography and functional brain mapping were performed using direct electrocortical stimulation sparing the motor cortex, with the objective of better defining the resection borders of the lesion. The lesion was located approximately 2 cm from the central sulcus. Intraoperative electrocorticography showed continuous polyspikes present over a 16-channel 4 × 4 subdural grid placed over the right premotor cortex with inter-electrode distances of 1 cm, and BIRDs characterized by a burst of spikes lasting more than 0.5 s and with higher amplitude over electrodes #9 and #10 (Fig. 2). The resected area corresponded to the position of the electrodes #9 and #10 because they showed atypical BIRDs, which led to the addition of non-lesional resection. The polyspikes were not considered for resection because they were too diffuse and were suspected of overlapping with the eloquent cortex (Fig. 2). Postoperative biopsy revealed pathology of FCD type IIa. The patient was seizure-free and antiseizure medication was gradually withdrawn by the 2-year follow-up. The patient remained on an IA Engel Class outcome at the 6-year follow-up.

## Discussion

ECoG is a readily available technique in developing countries for evaluating patients for epilepsy surgery, in contrast to the limited availability of long-term video-EEG monitoring due to a lack of access to EEG equipment, qualified technologists, infrastructure, and hospital beds [2]. FCD is associated with various patterns on interictal EEG and ECoG recordings, including continuous epileptiform discharges, rhythmic epileptiform discharges (type 1: trains of repetitive, rhythmic 4–10 Hz spikes/sharp waves lasting 1–4 s; type 2: quasi-continuous, slower, rhythmic 2–7 Hz sharp waves), focal polyspikes, frequent rhythmic bursting epileptiform activity, and repetitive epileptiform discharges [3]. Likewise, high-frequency oscillations on ECoG have been associated with a seizure-free outcome following complete resection [5, 6]; during frontal lobe seizures, rapid propagation occurs after seizure onset, often resulting in non-localized seizure onset and subsequently a poor surgical outcome [6, 7]. Low-voltage fast activity predicts favorable surgical outcomes in patients with frontal lobe epilepsies [8, 9]. The ictal pattern of low-voltage fast activity and gamma activity has been associated with seizure freedom after epilepsy surgery, while it is uncommon in epilepsy patients with recurrent seizures after surgery [5, 7].

In the present case, the lesion was localized by high-resolution, epilepsy-protocol brain MRI (3T). For lesional epilepsy, MRI is critical for the diagnosis and for determining the presurgical workup, leading to better outcomes. Emerging higher-strength magnets (i.e., 7T) offer a significantly greater resolution. However, they are not readily available in developing countries, which hinders the visualization of structural lesions, and requires the alternative use of intracranial EEG and ECoG [10]. The decision to move to ECoG can be made with high clinical suspicion of localization based on semiology, scalp EEG, and lesional and functional deficit zones [2]. In this case, we found atypical BIRDs on ECoG, which were also observed on scalp EEG.

BIRDs have been identified using scalp EEG in critically ill patients, and they are correlated with a higher risk of seizures, worse outcomes, and a higher likelihood of acute brain injury. In the non-critically ill population, BIRDs are associated with drug-resistant epilepsy and appear to have a value in localizing the seizure onset zone. In this case we found BIRDs on scalp EEG [11]. Intraoperative intracranial EEG recordings revealed similar features. Although even lesional focal epilepsies due to FCD are not 100% seizure-free, the finding of BIRDs raises the question of whether this biomarker may have the same clinical significance as BIRDs on scalp EEG in critically ill patients. Comparative trials are needed to answer this critical question. We noted some atypical features, suggesting flexible use of this terminology over the intensive care unit (ICU) EEG recordings to allow evolution and variability of this crucial finding.

Since BIRDs on intracranial EEG show similar frequencies as those of electroclinical seizures on scalp EEG and may potentially represent ictal recordings, we propose that intracranial EEG may frequently detect subclinical seizures [12, 13].

In this patient, surgical resection of the regions showing focal atypical BIRDs led to the elimination of the previously seen polyspikes when the subdural grid was placed immediately after the resection. No perioperative complications occurred. We speculate that localized atypical BIRDs on intraoperative ECoG may be a reliable biomarker for postsurgical outcomes [14].

To our knowledge, this is the first description of BIRDs as a part of an intraoperative ECoG-based interictal-ictal continuum in pathologically proven FCD IIa, in a patient with focal BIRDs on scalp EEG. Furthermore, this report has particular implications for developing countries where short-term video-EEG is used on patients with drug-resistant focal epilepsies to provide prolonged seizure-freedom outcomes after surgical resection. Further neurophysiologic studies are needed to better understand the value of BIRDs.

## Conclusions

We report the potential use of BIRDs on short-term intraoperative ECoG for localization purpose for successful epilepsy surgery. Our report is particularly relevant to others in developing countries where resources are limited. We extend the interictal-ictal continuum to include BIRDs recorded in the operating room and further emphasize its impact and disease-independent specificity.

## Data Availability

All data generated or analysed during this study are included in this published article.

## Abbreviations

BIRDs: Brief potentially ictal rhythmic discharges
ECoG: Electrocorticography
EEG: Electroencephalography
FCD: Focal cortical dysplasia
LVFA: Low-voltage ictal fast activity
MRI: Magnetic resonance imaging
